# Investigation and Comparison of Specific Antibodies’ Affinity Interaction with SARS-CoV-2 Wild-Type, B.1.1.7, and B.1.351 Spike Protein by Total Internal Reflection Ellipsometry

**DOI:** 10.3390/bios12050351

**Published:** 2022-05-18

**Authors:** Ieva Plikusiene, Vincentas Maciulis, Silvija Juciute, Ruta Maciuleviciene, Saulius Balevicius, Arunas Ramanavicius, Almira Ramanaviciene

**Affiliations:** 1 NanoTechnas—Center of Nanotechnology and Materials Science, Faculty of Chemistry and Geosciences, Vilnius University, Naugarduko Str. 24, 03225 Vilnius, Lithuania; ieva.plikusiene@chgf.vu.lt (I.P.); vincentas.maciulis@ftmc.lt (V.M.); silvija.juciute@chgf.stud.vu.lt (S.J.); ruta.maciuleviciene@gmail.com (R.M.); saulius.balevicius@ftmc.lt (S.B.); arunas.ramanavicius@chf.vu.lt (A.R.); 2 State Research Institute Center for Physical and Technological Sciences, Sauletekio Ave. 3, 03225 Vilnius, Lithuania

**Keywords:** total internal reflection ellipsometry, SARS-CoV-2, immune complex, antibody–antigen interaction

## Abstract

SARS-CoV-2 vaccines provide strong protection against COVID-19. However, the emergence of SARS-CoV-2 variants has raised concerns about the efficacy of vaccines. In this study, we investigated the interactions of specific polyclonal human antibodies (pAb-SCoV2-S) produced after vaccination with the Vaxzevria vaccine with the spike proteins of three SARS-CoV-2 variants of concern: wild-type, B.1.1.7, and B.1.351. Highly sensitive, label-free, and real-time monitoring of these interactions was accomplished using the total internal reflection ellipsometry method. Thermodynamic parameters such as association and dissociation rate constants, the stable immune complex formation rate constant (*k*_r_), the equilibrium association and dissociation (*K*_D_) constants and steric factors (*P*_s_) were calculated using a two-step irreversible binding mathematical model. The results obtained show that the *K*_D_ values for the specific antibody interactions with all three types of spike protein are in the same nanomolar range. The *K*_D_ values for B.1.1.7 and B.1.351 suggest that the antibody produced after vaccination can successfully protect the population from the alpha (B.1.1.7) and beta (B.1.351) SARS-CoV-2 mutations. The steric factors (*P*_s_) obtained for all three types of spike proteins showed a 100-fold lower requirement for the formation of an immune complex when compared with nucleocapsid protein.

## 1. Introduction

One of the key factors in preventing the spread of coronavirus disease 2019 (COVID-19), caused by severe acute respiratory syndrome coronavirus 2 (SARS-CoV-2), is the ability of the human immune system to produce neutralizing antibodies that could successfully block the SARS-CoV-2 spike (S) protein (in specific regions) and prevent its binding to the angiotensin-converting enzyme 2 (ACE2) receptor. However, the changes in SARS-CoV-2 over time, specifically in the S protein and some occurring mutations, are associated with viral survival capability, the rate of virus spread, and the severity of the disease. Additionally, these mutations in SARS-CoV-2 could affect the effectiveness of vaccines, therapeutic drugs, and diagnostic tools, as well as posing an increased risk to global public health. The World Health Organization (WHO), in collaboration with partners, has been monitoring and evaluating the evolution of the wild-type SARS-CoV-2, identified in Wuhan, China, since January 2020. Since then, an emergence of mutations has been recorded and compared to the reference SARS-CoV-2 genome. The WHO-convened expert group recommended the use of letters of the Greek alphabet (i.e., alpha, beta, gamma, delta, etc.) to name the SARS-CoV-2 variants [1].

The S protein is a large transmembrane homotrimer located on the surface of the virus. Each monomer is composed of two subunits, namely S1—with the receptor-binding domain (RBD) being responsible for binding with the ACE2 receptor present on the cell surface, and S2—responsible for the fusion of viral and host membranes [2,3]. Alpha SARS-CoV-2 (B.1.1.7) was first detected in the UK in autumn 2020. This variant is characterized by a large number of mutations, particularly within the S protein, as well as its being easier and faster to spread than other variants, mainly due to a single amino acid change (N501Y) in the RBD of the S protein [4]. The N501Y mutation improved the affinity of RBD for the human ACE2 receptor [5,6]. Almost all currently available vaccines aim to increase immunity specifically against the spike protein or just parts of it, mainly the RBD [7]. Several vaccine-producing companies, including Pfizer–BioNTech, Moderna, Astra Zeneca–Oxford, and others, confirmed the efficacy of their vaccines against alpha SARS-CoV-2. Beta SARS-CoV-2 (B.1.351) was first detected in South Africa (the genome of a variant strain was published in December 2020) and can also be characterized by specific mutations in the RBD of its S protein (N501Y, K417N, and E484K). These mutations enhance the affinity of the RBD for ACE2, while the E484K mutation could lead to an evasion of the immune system’s response [8]. It is of paramount importance to understand the impact of SARS-CoV-2 mutations on the changes in viral infectivity and transmission. It is also relevant to clarify the role of mutations in the S protein, in terms of its interactions with antibodies produced in healthy (i.e., without natural exposure to SARS-CoV-2) and vaccinated individuals. The spread of the SARS-CoV-2 variants of concern is a challenge for the scientific community, who seek to be able to predict the effects of different mutations on both the virus’s binding to the receptor and the antibody’s binding to the virus [9]. It is well known that antibodies produced by the humoral response of the immune system after vaccination are one of the key factors in overcoming SARS-CoV-2, and their affinity for viral antigens is of high importance. However, there is still a lack of information on the antibodies’ protection against new emerging variants of SARS-CoV-2, such as the alpha (B.1.1.7) and beta (B.1.351) variants that arose from the United Kingdom and South Africa, respectively. It was reported that B.1.1.7 still remains sensitive to neutralization with antibodies [10]. The results of neutralizing activity induced by a vectored vaccine against the B.1.1.7 and B.1.351 strains demonstrated that widespread immune escape was not observed in B.1.1.7, but greater resistance to B.1.351 was indicated in both the pseudo-virus and the live-virus neutralization assays [11]. Additionally, serological assays are mainly based on the wild-type SARS-CoV-2 S protein. However, the sensitive and specific assays used to monitor the antibody’s immune response profile against four viral antigens (typically the RBD, the nucleocapsid, and the S1 and S2 proteins) were developed by Bio-Rad Laboratories, Inc. [12]. Thus, the spectrum of humoral responses can be assessed after COVID-19 infection or over time after vaccination.

The affinity of a specific antibody to viral antigens shows how effectively the immune complex is formed. Furthermore, the association and dissociation rate constants can give us important information about the thermodynamic parameters of this process. It was demonstrated that thermodynamic parameters enable a quantitative evaluation of an antibody’s specific ability to inhibit the binding of the viral spike protein for the ACE2 receptor located in the host cell [13]. The affinity of a specific antibody for the SARS-CoV-2 S protein or for the ACE2 receptor can be measured using a sensitive, label-free, and real-time monitoring optical surface plasmon resonance (SPR) method [14,15,16]. Despite the fact that SPR is common in many laboratories, the other optical method, spectroscopic ellipsometry in total internal reflection mode (TIRE), recently attracted a high level of interest for its application in antibody–antigen interaction measurements, due to its higher sensitivity than SPR [17,18,19,20,21,22]. During an experiment using TIRE, the light wave polarization state’s change upon the reflection from the surface is measured [19]. The TIRE method is capable of detecting even small changes in the ambient refractive index caused by the immobilization of antigens or antibodies and the interaction between them on the surface [23,24,25]. During TIRE measurements, two ellipsometric parameters are determined: *Ψ* corresponds to the light wave amplitude and *Δ* to the light wave phase shift after the light reflection from the surface. The change in the ellipsometric parameter *Ψ* is sensitive to the large concentration range of biomolecules during the formation of the layer. However, the sensitivity of the parameter *Δ* decreases significantly due to the shift in surface plasmon resonance if the concentration of biomolecules is high [18]. Therefore, the measurement and assessment of both ellipsometric parameters *Δ* and *Ψ* enable a more detailed evaluation of interacting biomolecules’ affinity and association/dissociation rate constants.

In this study, we performed a highly sensitive, label-free, and real-time TIRE monitoring of the interaction kinetics between the specific polyclonal human antibody (pAb-SCoV2-S) produced after vaccination and the covalently immobilized spike proteins of the three SARS-CoV-2 variants of concern—wild-type (SCoV2-S), B.1.1.7 or so-called alpha (SCoV2-αS), and B.1.351 or so-called beta (SCoV2-βS). Here, we reported the comparison of the thermodynamic parameters, such as the association rate constant (*k*_a_), the dissociation rate constant (*k*_d_), the stable immune complex formation rate constant (*k*_r_), the equilibrium association and dissociation constants (*K*_A_, *K*_D_), and steric factors (*P*_s_), calculated using a two-step irreversible binding mathematical model.

## 2. Materials and Methods

### 2.1. Materials

N-(3-dimethylaminopropyl)-N′-ethyl-carbodiimide hydrochloride (EDC) and N-hydroxysuccinimide (NHS) were purchased from Merck Millipore (USA). Ethanolamine, sodium dodecyl sulphate, sodium hydroxide, and 11-Mercaptoundecanoic acid (11-MUA) were purchased from Sigma Aldrich. The SPR sensors’ disc, coated with a 46 nm gold layer, was received from XanTech bioanalytics (Duesseldorf, Germany). The three types of SARS-CoV-2 recombinant S proteins—the wild-type (SCoV2-S), the alpha variant (SCoV2-αS: contains nine mutations compared to the reference SARS-CoV-2 S protein), and the beta variant (SCoV2-βS: contains ten mutations compared to the reference SARS-CoV-2 S protein)—expressed in yeast Saccharomyces cerevisiae (purity > 85%), were purchased from Baltymas (Vilnius, Lithuania).

Human blood serum was collected from two volunteers. One serum was obtained from the volunteer vaccinated using 1 dose of the Vaxzevria vaccine 3 weeks before the experiment, whilst the other (control serum) was obtained from a healthy volunteer before the pandemic. Whole blood was collected in a vacutainer test tube containing 3.5 mL of CAT serum separator clot activator (Greiner Bio-One GmbH, Kremsmünster, Austria), in the laboratory of Tavo Klinika, LtD (Vilnius, Lithuania). Serum was obtained after centrifugation at 5000× *g* for 15 min. The titer of the antibody against the S protein of SARS-CoV-2 (pAb-SCoV2-S) was obtained using a chemiluminescent microparticle immunoassay. The titer was then recalculated to a molar concentration, and the primary blood serum pAb-SCoV2-S concentration was obtained at 33.96 nM [26]. Blood serum dilutions with a phosphate-buffered saline (PBS) solution at pH 7.4 (Carl Roth, Karlsruhe, Germany) and at 1:4, 1:10, 1:20, 1:30, and 1:40 ratios were used for the kinetics measurements of the pAb-SCoV2-S interaction with the three types of S protein. The serum sample was stored at − 20 °C until analysis. The sample was collected in accordance with the Lithuania ethics law. This study did not need the approval of the ethics committee (confirmed by the Vilnius Regional Biomedical Research Ethics Committee).

### 2.2. Modification of the Gold-Coated SPR Sensor Disc with SCoV2-S, SCoV2-αS, or SCoV2-βS

**Figure 1:**
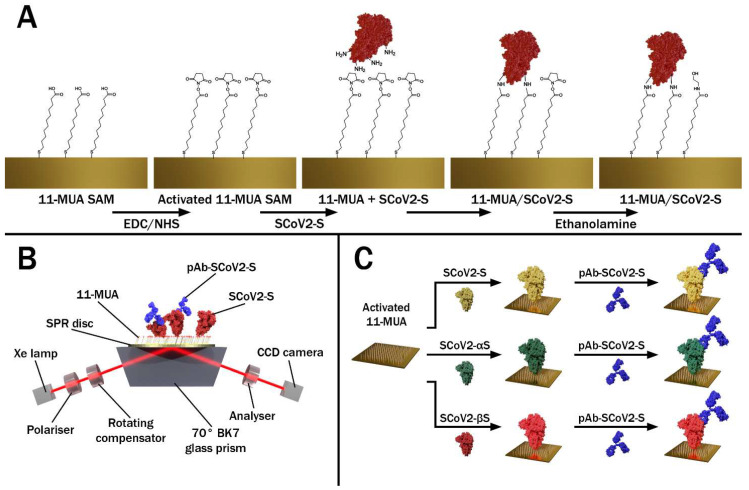
(**A**) Schematic representation of SARS-CoV-2 S protein (SCoV2-S, SCoV2-αS, or SCoV2-βS) covalent immobilization on the gold-coated SPR sensor disc pre-modified with 11-MUA SAM. (**B**) The principle scheme representing total internal reflection ellipsometry measurements. (**C**) Schematic representation of SCoV2-S, SCoV2-αS, and SCoV2-βS covalent immobilization and interaction with specific polyclonal antibodies (pAb-SCoV2-S).

The covalent immobilization of SCoV2-S, SCoV2-αS, or SCoV2-βS on the gold-coated SPR sensor disc’s surface was performed under standard protocol using an 11-MUA self-assembled monolayer (SAM) [21,22,27,28,29]. Briefly, the gold-coated SPR sensor disc was rinsed with hexane and methanol and then immersed in methanol for 3 min for ultrasound treatment. Once dried, the SPR sensor disc was immersed in a 1 mM solution of 11-MUA in methanol for 18 h to form an 11-MUA SAM. Then, the activation of the 11-MUA SAM carboxyl groups required for the covalent immobilization of SCoV2-S, SCoV2-αS, or SCoV2-βS was accomplished by injecting the solution consisting of 0.1 M NHS and 0.4 M of EDC, mixed in equal parts, into a TIRE measurement chamber for 15 min. After the activation of the carboxyl groups, the TIRE chamber was rinsed with a PBS solution at a pH level of 7.4. Then, 333 nM SCoV2-S diluted in the PBS was injected into the TIRE chamber and incubated for 60 min. After rinsing with the PBS solution, the surface was exposed to 1 M ethanolamine at a pH level of 8.5 for 10 min to deactivate any active carboxyl groups present in the 11-MUA SAM. The schematic representation of all the steps in the surface modification is presented in Figure 1A. The same immobilization protocol was applied for SCoV2-αS and SCoV2-βS using the same 333 nM concentration in PBS (Figure 1C).

### 2.3. TIRE Measurements

The ellipsometric measurements were conducted using a rotating compensator ellipsometer J. A. Woollam M2000X (Lincoln, NE, USA). All TIRE experiments were carried out at a 70° angle of incident in the spectral range of 200–1000 nm. A BK7 glass prism (set at 70°) was connected to the commercial SPR sensor disc via a refractive index matching fluid and mounted on the TIRE chamber with a 5 mm diameter. In the TIRE experimental setup, a liquid handling system with a custom-built Teflon chamber was used for measurements in liquids, and this schematic measurement setup is presented in Figure 1B. After each step in the surface modification, the spectra of the ellipsometric parameters *Ψ* and *Δ* vs. *λ* were recorded and compared with those obtained previously. Here, we present only the evolution in time of parameter *Ψ*, as this ellipsometric parameter sensitivity remains the same throughout a wide concentration range.

## 3. Results and Discussion

### 3.1. Covalent Immobilization of SCoV2-S, SCoV2-αS, or SCoV2-βS on 11-MUA SAM

**Figure 2:**
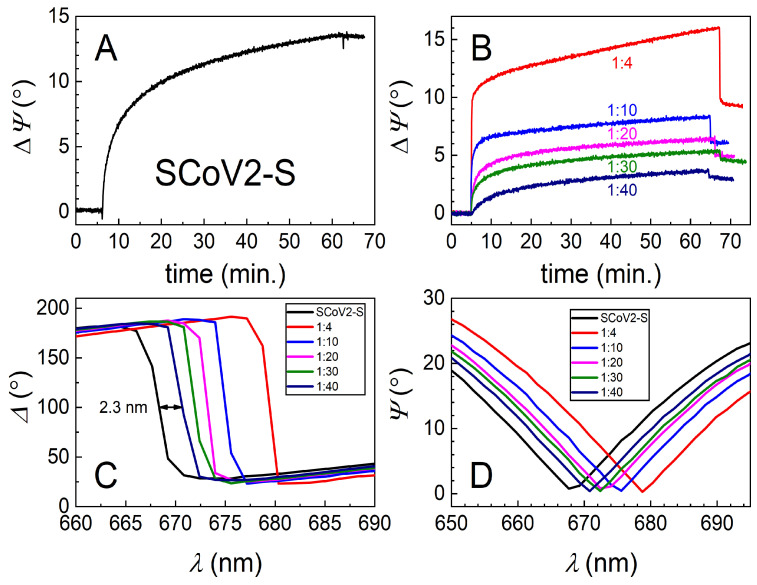
Time-resolved TIRE kinetics and ellipsometric parameters of SCoV2-S immobilization and interaction with specific polyclonal antibodies. (**A**) Kinetics of covalent SCoV2-S immobilization on 11-MUA SAM modified gold-coated SPR sensor disc; (**B**) kinetics of polyclonal antibody interaction with covalently immobilized ScoV2-S at different serum dilutions (1:4, 1:10, 1:20, 1:30, and 1:40); (**C**) *Δ* and (**D**) *Ψ* spectral shift after immune complex formation using the same dilutions of polyclonal antibodies.

**Figure 3:**
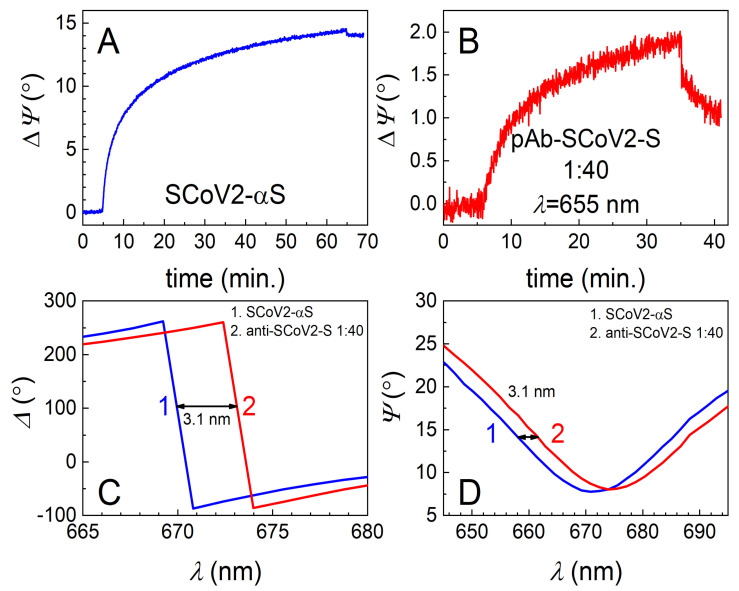
Time-resolved TIRE kinetics and ellipsometric parameters of SCoV2-αS immobilization and interaction with specific polyclonal antibodies. (**A**) Kinetics of covalent SCoV2α-S immobilization on 11-MUA SAM modified gold-coated SPR sensor disc; (**B**) kinetics of polyclonal antibody interaction with covalently immobilized SCoV2-αS at 1:40 dilution of serum; (**C**) *Δ* and (**D**) *Ψ* spectral shift after immune complex formation using the same dilutions of polyclonal antibodies.

**Figure 4:**
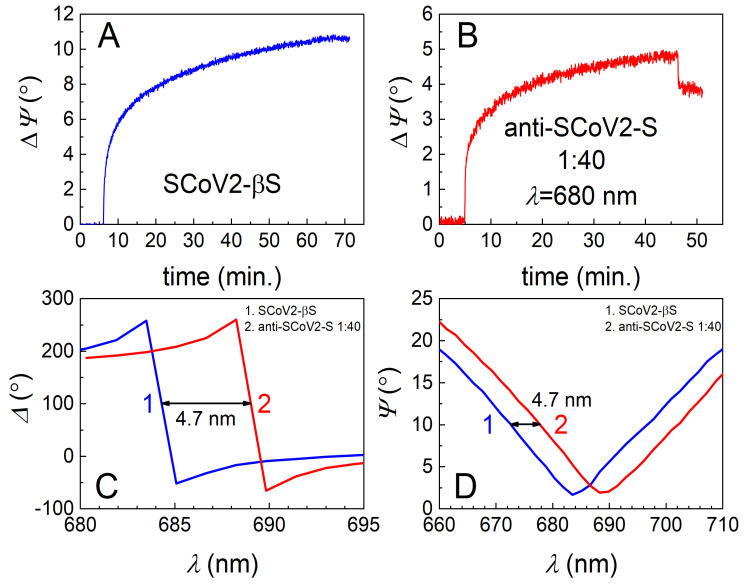
Time-resolved TIRE kinetics and ellipsometric parameters of SCoV2-βS immobilization and interaction with specific polyclonal antibodies. (**A**) Kinetics of covalent SCoV2β-S immobilization on 11-MUA SAM modified gold-coated SPR sensor disc; (**B**) kinetics of polyclonal antibody interaction with covalently immobilized SCoV2-βS at 1:40 dilution of serum; (**C**) *Δ* and (**D**) *Ψ* spectral shift after the formation of immune complexes using the same dilutions of polyclonal antibodies.

The TIRE method was used to study the covalent immobilization of three different mutations, or so-called variants of concern (VOCs), of the SARS-CoV-2 S protein and for the evaluation of interaction kinetics with diluted human blood serum containing specific pAb-SCoV2-S antibodies. The 333 nM of SCoV2-S, SCoV2-αS, and SCoV2-βS in the PBS solution were covalently immobilized on the activated 11-MUA SAM according to the protocol described in the Materials and Methods part. All three injected solutions of the S protein were kept in the chamber for about 55–60 min, and then washed with PBS. Covalent immobilization kinetics were analyzed at 661 nm for SCoV2-S, 656 nm for SCoV2-αS, and 670 nm for SCoV2-βS, close to the SPR dip. As can be seen in Figure 2A, Figure 3A, and Figure 4A, the ellipsometric parameter *ΔΨ* change was 13.5° for SCoV2-S, 14.0° for SCoV2-αS, and 11.0° for SCoV2-βS after immobilization, respectively.

### 3.2. Immune Complex Formation between Covalently Immobilized SCoV2-S, SCoV2-αS, or SCoV2-βS and Specific Polyclonal Antibodies

For the evaluation of the affinity interaction and the formation of immune complexes between immobilized SCoV2-S, SCoV2-αS, or SCoV2-βS and pAb-SCoV2-S antibodies, human blood serum was diluted with PBS at 1:4, 1:10, 1:20, 1:30, and 1:40 ratios. First, the solution containing 1:40 diluted human blood serum with the 0.849 nM concentration of pAb-SCoV2-S was injected into the TIRE chamber. After 60 min, the chamber was washed with PBS. In the next step of the experiment, the surface was washed with the regeneration solution consisting of 17.34 mM SDS and 10 mM NaOH for 1 min, to remove the affinity-bound antibodies from the surface. Then, an additional rinsing with PBS was performed. The same experiments were performed with all previously mentioned blood serum dilutions at 1:30, 1:20, 1:10 and 1:4 ratios. The blood serum collected before the start of the COVID-19 pandemic and diluted at a 1:40 ratio with PBS was used to evaluate the non-specific interaction of blood serum with the covalently immobilized S protein VOCs. The time-resolved TIRE kinetics and the ellipsometric parameters of the covalently immobilized SCoV2-S interaction with specific pAb-SCoV2-S are presented in Figure 2B.

The highest concentration of the pAb-SCoV2-S antibody in the serum sample (with a dilution ratio at 1:4 or with an 8.49 nM concentration) showed a 16° change in the ellipsometric parameter *Ψ* after 60 min, while after interaction with the sample of the lowest concentration of the pAb-SCoV2-S antibody (with a dilution ratio at 1:40 or with a 0.849 nM), it was 3.8° (Figure 2B). The *Ψ* changes obtained using SCoV2-S, SCoV2-αS, and SCoV2-βS are presented (Figure 2B, Figure 3B, and Figure 4B) as eliminating the signal (*ΔΨ* = 2.5°) that results after a non-specific interaction of serum without antibodies against the SARS-CoV-2 S protein with the immobilized S protein VOC. As can be seen, the TIRE method is able to detect a small concentration (0.849 nM) of the pAb-SCoV2-S antibody in blood serum and is also able to monitor such interaction kinetics (Figure 2B, at a 1:40 ratio). The spectral shift of the ellipsometric parameters *Δ* and *Ψ* vs. *λ* was 2.3 nm after the formation of the SCoV2-S/pAb-SCoV2-S immune complex, using blood serum diluted at a 1:40 ratio (Figure 2C,D). For analogous experiments with covalently immobilized SCoV2-αS or SCoV2-βS proteins, we chose to use the same 0.849 nM concentration (with a dilution ratio at 1:40) of the pAb-SCoV2-S antibody. After 60, 35, and 45 min, the change in the ellipsometric parameter *ΔΨ* was still significant, and washing with PBS showed only a small signal drop (Figure 2B, Figure 3B and Figure 4B).

For the investigation into the immune complex formation between SCoV2-αS and pAb-SCoV2-S, SCoV2-αS was covalently immobilized on the 11-MUA SAM, and this immobilization kinetics was recorded using TIRE (Figure 3A). During the interaction between the covalently immobilized SCoV2-αS with a 0.849 nM concentration of pAb-SCoV2-S antibodies (the formation of the SCoV2-αS/pAb-SCoV2-S immune complex), the change in the ellipsometric parameter *ΔΨ* was 2.01°, and the spectral shift of *Δ* and *Ψ* vs. *λ* was 3.1 nm to longer wavelengths. Furthermore, the covalent immobilization of SCoV2-βS on the 11-MUA SAM was performed, and immobilization kinetics was recorded using TIRE (Figure 4A). The immune complex formation kinetics, using the same pAb-SCoV2-S antibody concentration (0.849 nM), and the covalently immobilized SCoV2-βS was recorded. After the immune complex formation, the change in *ΔΨ* was 4.96° (Figure 4B), and a 4.7 nm spectral shift in *Δ* and *Ψ* vs. *λ* (Figure 4C,D) was observed.

**Figure 5:**
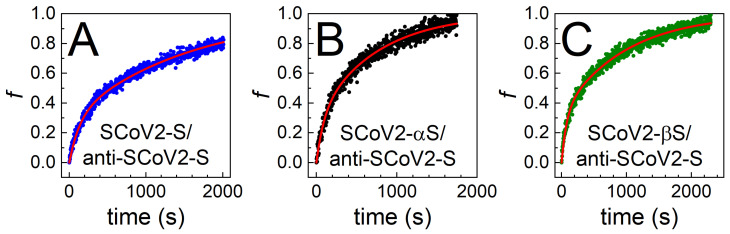
Normalized pAb-SCoV2-S antibody surface concentration (*f*) evolution in time obtained using 1:40 diluted blood serum during the formation of the immune complex with (**A**) SCoV2-S, (**B**) SCoV2-αS, (**C**) SCoV2-βS. Points correspond to experimentally obtained results, while lines for fitting are derived by using two-step irreversible binding immune complex formation mathematical modeling.

As can be seen from the covalent SCoV2-S, SCoV2-αS, and SCoV2-βS immobilization experiments using TIRE (Figure 2A, Figure 3A, and Figure 4A), changes in the ellipsometric parameter *Ψ* after 60 min were similar. These results indicate that the three types of SARS-CoV-2 S protein VOCs can be covalently immobilized on the sensing surface successfully, using the 11-MUA SAM. The SCoV2-S, SCoV2-αS, and SCoV2-βS interactions with pAb-SCoV2-S and the immune complexes formation kinetics were analyzed by two-step irreversible binding mathematical modeling [21]. Understanding the mechanism of specific antibody interaction with the whole SARS-CoV-2 S protein is critical for the development of effective therapeutics, vaccines, and detection tools [30]. Therefore, it is very important to obtain as much information as possible on the formation of immune complexes between the S protein variants of SCoV2-S, SCoV2-αS, or SCoV2-βS and the specific antibodies formed during the humoral response of the immune system. The antibodies that bind outside of the RBD region stimulate the antiviral activity of the immune system and can provide protection against COVID-19, effectively turning this serious illness into a milder form, such as the common cold [31]. Other authors reported their structural analysis of neutralizing antibodies that were RBD-directed and N-terminal (NTD)-directed. It was shown that in opposit to RBD-directed antibodies that recognize multiple non-overlapping epitopes, NTD-directed antibodies target a single supersite of an S protein with high affinity, in the range of 2–70 nM [32]. In addition to our experimental results, we used a two-step irreversible binding mathematical model [21,27] for the evaluation of interaction kinetics between covalently immobilized SCoV2-S, SCoV2-αS, SCoV2-βS, and a specific polyclonal antibody obtained from a volunteer’s blood serum, who was vaccinated with one dose of the Vaxzevria vaccine, which eliminated non-specific interactions. The two-step irreversible binding mathematical model, which describes the formation of an immune complex, was applied in our previous work on the interaction of a nucleocapsid protein with a polyclonal antibody [33]. In the fitting of experimentally obtained kinetics, a normalized surface concentration allowed us to calculate the association rate constant (*k*_a_), the dissociation rate constant (*k*_d_), the stable immune complex formation rate constant (*k*_r_), equilibrium association and dissociation constants (*K*_A_, *K*_D_), and steric factors (*P*_s_). The fitting results obtained via this mathematical model of irreversible two-step antibody binding to covalently immobilized SCoV2-S, SCoV2-αS, or SCoV2-βS are presented in Figure 5. 

The calculated thermodynamic parameters of immune complex formation can provide a deeper understanding on how a specific antibody from human blood serum can interact with the wild-type (SCoV2-S) and the mutated (SCoV2-αS and SCoV2-βS) SARS-CoV-2 S proteins. The calculated values for the pAb-SCoV2-S immune complex formation with the three types of investigated S protein are presented in Table 1.

The results obtained show that the *K*_D_ for the specific antibody interaction with the mutated spike protein SCoV2-αS and SCoV2-βS are in the same range of nM, and also demonstrate the high antibody affinity against these mutations. Moreover, the *K*_D_ value for the antibodies produced against the target protein SCoV2-S is lowest (*K*_D_ = 3.22 × 10^−10^ M). However, the nM range of the *K*_D_ value for SCoV2-αS and SCoV2-βS suggests that the antibody produced after vaccination can successfully protect the population from the SARS-CoV-2 alpha (B.1.1.7) and beta (B.1.351) mutations. Other authors also reported that high affinity was observed of monoclonal anti-RBD neutralizing antibodies for RBD [34]. As it was shown by the group of authors, mutations in the RBD of the alpha (B.1.1.7) and beta (B.1.351) increased the affinity to the ACE2 receptor [6,15,35]. It was reported that affinity values varied from *K*_D_ 6 to 133 nM [15].

The obtained stable immune complex formation rate constants *k*_r_ are in the same range for SARS-CoV-2 alpha (B.1.1.7) and beta (B.1.351). In the present study, we calculated steric factors *P*_s_ for all three types of immune complexes studied: SCoV2-S/pAb-SCoV2-S, SCoV2-αS/pAb-SCoV2-S, and SCoV2-βS/pAb-SCoV2-S. The *P*_s_ provides information about the steric requirements for an antibody and antigen to form a stable complex successfully and can be calculated from the encounter theory [36,37]. We calculated *P*_s_ in our previous work on the covalently immobilized SARS-CoV-2 nucleocapsid protein and the specific antibody immune complex formation, and the obtained value was 5.49 × 10^−4^. When comparing the *P*_s_ values obtained for nucleocapsid and SCoV2-S, SCoV2-αS, and SCoV2-βS, it is evident that specifically pAb-SCoV2-S has a 100-fold lower requirement for the formation of an immune complex. This can be explained by the fact of immune complexes being able to form more easily with larger antigens. In this case, the mass of the S protein (trimer) is higher (monomer is 114.11 kDa) compared to the nucleocapsid (49 kDa).

## 4. Conclusions

In the present investigation, we demonstrated how the highly sensitive TIRE method can be applied for the evaluation of interaction kinetics between the covalently immobilized SARS-CoV-2 S protein variants of concern and the specific polyclonal antibody from diluted voluntary blood serum. The calculated equilibrium dissociation constants *K*_D_ for the pAb-SCoV2-S immune complex formation with the covalently immobilized SCoV2-S, SCoV2-αS, and SCoV2-βS showed that these antibodies, developed by the humoral response of the immune system after vaccination, are able to bind to all three types of the mutated S protein with high affinity. The calculations of the steric factors showed that pAb-SCoV2-S can form an immune complex easily enough in comparison to the immune complex formation with a nucleocapsid protein. These results show the ability of the antibody produced after vaccination with Vaxzevria to protect the population from the SARS-CoV-2 mutation alpha (B.1.1.7) and beta (B.1.351).

## Figures and Tables

**Table 1:** Thermodynamic parameters of specific antibody interaction with variants of concern.

	SCoV2-S	SCoV2-αS	SCoV2-βS
***k*_a_ (M^−1^s^−1^)**	3.19 × 10^6^	5.01 × 10^6^	5.92 × 10^6^
***k*_d_ (s^−1^)**	1.03 × 10^−5^	1.97 × 10^−5^	3.27 × 10^−5^
***K*_A_ (M^−1^)**	3.09 × 10^11^	2.54 × 10^11^	1.81 × 10^11^
***K*_D_ (M)**	3.22 × 10^−10^	3.93∙10^−10^	5.47 × 10^−10^
***k*_r_ (s^−1^)**	5.05 × 10^−6^	1.48 × 10^−5^	1.33 × 10^−5^
***P*_s_**	1.51 × 10^−2^	1.88 × 10^−2^	2.01 × 10^−2^

Association rate constant (*k*_a_), dissociation rate constant (*k*_d_), stable immune complex rate constant (*k*_r_), equilibrium association constant (*K*_A_), equilibrium dissociation constant (*K*_D_), and steric factors (*P*_s_).

## Data Availability

The data presented in this study are available on request from the first author.
